# Identifying extractable profiles from 3D printed medical devices

**DOI:** 10.1371/journal.pone.0217137

**Published:** 2019-05-22

**Authors:** Joel D. Rindelaub, Zane Baird, Bruce A. Lindner, Angela A. Strantz

**Affiliations:** 1 School of Chemical Sciences, University of Auckland, Auckland, NZ; 2 Train Industries, Bloomington, Indiana, United States of America; 3 Pace Analytical Life Sciences, Oakdale, Minnesota, United States of America; University of Pennsylvania, UNITED STATES

## Abstract

With the ability to create customizable products tailored to individual patients, the use of 3D printed medical devices has rapidly increased in recent years. Despite such interest in these materials, a risk assessment based on the material characterization of final device extracts—as per regulatory guidance—has not yet been completed, even though the printing process may potentially impact the leachability of polymer components. To further our understanding of the chemical impact of 3D printed medical devices, this study investigated the extractable profiles of four different materials, including a PLA polymer advertised as “FDA-approved”. The fusion deposition modeling (FDM) printing process created distinct chemical and physical signatures in the extracts of certain materials. The application of an annealing procedure to printed devices led to a substantial decrease in extractable components by as much as a factor of 50. In addition, the use of a brass printing nozzle led to an increase in the amount of Pb detected in 3D printed device extracts. The data generated provides valuable information that can be used to help assess extractable risks of 3D printed medical devices, assist with future 3D printing designs, and may provide insight for agencies tasked with governing 3D printed medical device regulations.

## Introduction

The use of 3D printed medical devices is a growing industry across the globe, thanks to product customizability, short lead times, cost efficiency, and the ability to create unique devices not possible with traditional manufacturing techniques [1]. In addition to the fabrication of implants and patient models, 3D printing–also known as additive manufacturing–has potential for organ and tissue construction [2–6] as well as drug discovery, delivery, and dosage [7–9]. With a wide variety of beneficial applications, the 3D printing of medical materials is expected to reach a near $2 billion industry by 2024 [10].

As of 2015, at least 85 additive manufacturing devices have been approved by the U.S. Food and Drug Administration (FDA), with applications that include hearing aids, cranial plates, facial implants, dental crowns, spinal cages, and other implantable materials [1,11–13]. In the operating room, the use of custom-made materials has facilitated more efficient and successful surgeries, resulting in better patient outcomes [14–17].

FDA approval of 3D printed devices has largely been based on biocompatibility studies, specifically, an *in vitro* cytotoxicity test is used to investigate the biological reactivity of mammalian cell lines in contact with test articles or test article extracts [18]. However, information gained from cytotoxicity testing has several limitations, including challenges associated with describing long-term effects of a given exposure, evaluating the toxicity to an individual patient, and data interpretation from both inconsistent analysis methods and excessively toxic results [19]. Also, since damaged cells may remain fully functional for an appreciable amount of time before expiry, measuring cytotoxicity based on cellular function may underestimate total cytotoxicity [19]. Complete biocompatibility testing is recommended to also include sensitization, irritation, acute systemic toxicity, subacute toxicity, genotoxicity, implantation, and hemocompatibility, depending on the application of the device [20].

Despite this knowledge, many FDA approved submissions have demonstrated “substantial equivalence” to predicate devices through the 501(k) pathway without a chemical comparison of final device extracts [21]. Since there is a lack of chemical data on the impact of 3D printing processes, a large uncertainty in the actual equivalency of such an assessment still exists.

To fully understand the potential leachable profiles of medical devices, regulatory recommendations typically call for material characterization when requesting product approval [20,22], which can be accomplished via extractable/leachable (E&L) studies. The term leachable refers to a compound that migrates from a medical product during its intended use while an extractable is any compound that can be solvent or thermal extracted from a product or test article under controlled laboratory conditions [23]. As such, extractables identified are potential leachables. By generating analytical data that includes both the chemical identification and quantitative/semi-quantitative information of extractable components, E&L studies provide information needed to evaluate both the toxicity and exposure hazard of individual compounds [24].

Extractable/leachable components can originate from any part of the manufacturing process or from any post-processing treatment or container system [25]. Since the fusion deposition modelling (FDM) printing process, which is a common technique for 3D printing in the medical field [1,26], involves the application of high heat and contact with printer components, there may be an increased risk of polymer additive leaching, due to induced changes in the polymer’s crystallization, as well as potential chemical contamination from the printer itself.

To our knowledge, there currently are not any publicly available studies investigating the extractable profiles of FDM printed medical devices. Thus, four different FDM printed polymeric materials (polylactic acid, FDA-approved polylactic acid, polycarbonate, polyethylene terephthalate glycol, and polycarbonate) were extracted using solvents of varying polarity and analyzed for organic, elemental, and particulate matter composition. Results generated provide insight into how the 3D printing process can affect the chemical and physical properties of medical devices.

## Materials and methods

Extractable testing was performed on four different 3D printed polymers: polylactic acid (PLA), FDA-approved polylactic acid (FDA PLA), polyethylene terephthalate glycol (PETG), and polycarbonate (PC). It is important to note that while one of the PLA materials was marketed as “FDA-approved”, this term only corresponds to the use of an FDA compliant resin (as per the manufacturer), and does not actually hold approval from the U.S. Food and Drug Administration. Since the 3D printed devices in this study were not made for a specified medical treatment, a true leachable study under simulated use conditions was not possible, and, as such, only an extractable study was investigated.

**Fig 1:**
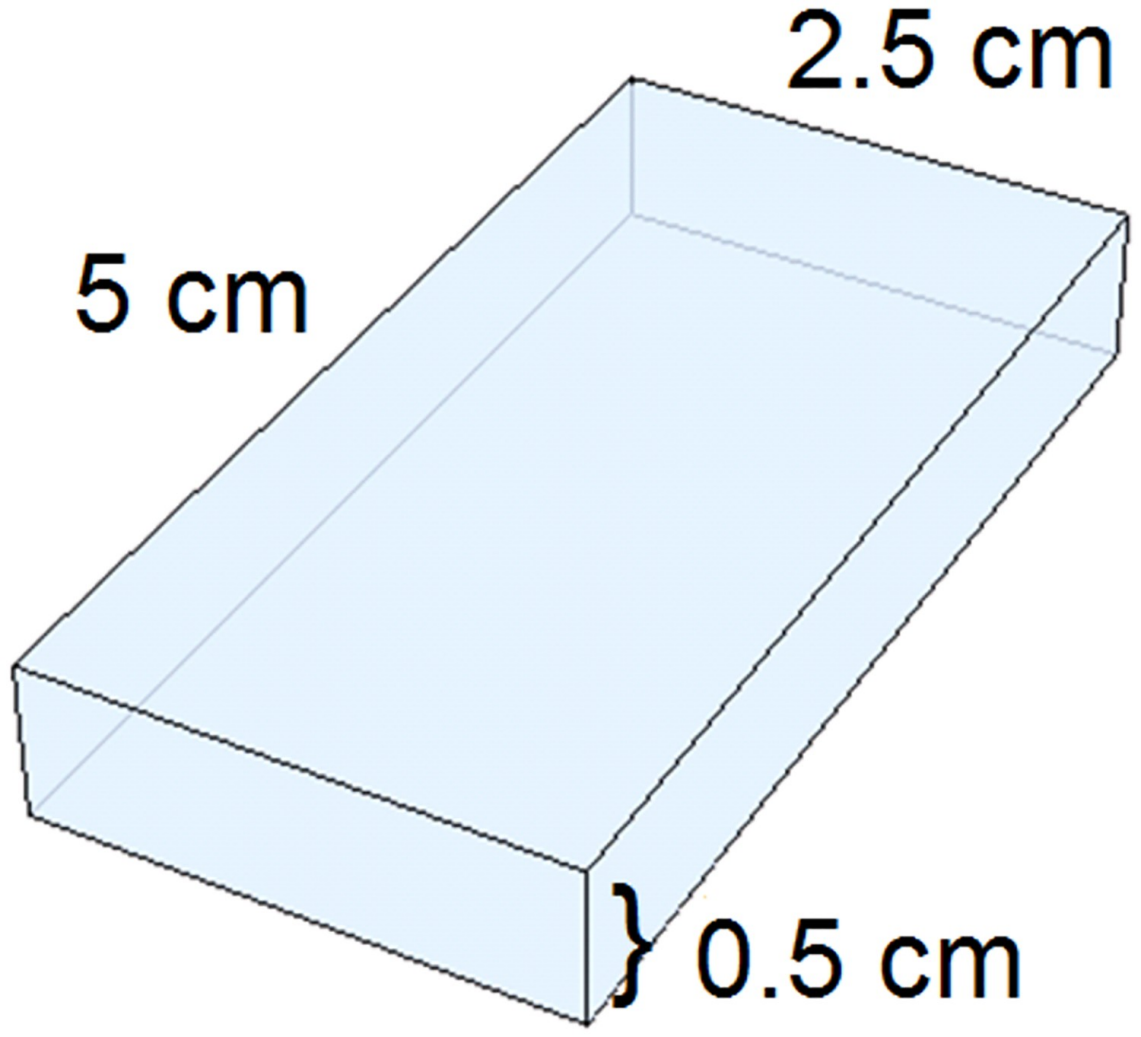
3D printed devices. A schematic of the final printed devices.

Rectangular 3D printed coupons were created from each polymer to represent medical devices, as per FDA recommendations [22], and will further be referred to as “devices” within this paper. The final printed devices were identical blocks with flat sides having dimensions of 5.0 x 2.5 x 0.5 cm for a surface area of 32.5 cm^2^. A description of the polymers chosen is given in Table 1, and a schematic of the devices is shown in Fig 1.

**Table 1:** Information regarding the polymer materials and 3D printing parameters used.

Polymer Details						Printing Parameters			
Material	Description	Manufacturer	Uses	Color	Mass (g)	Nozzle material	Bed Temp. (°C)	Nozzle Temp. (°C)	Extrusion multiplier
PLA	Polylactic acid	Makergeeks	Implantables	White	5.3	Stainless Steel	70	230	0.91
FDA PLA	FDA-approved Polylactic acid	Makergeeks	Implantables	Red	5.0	Brass	70	230	0.90
PETG	Polyethylene terephthalate-glycol	Colorfabb	Medical Device Packaging	Yellow	5.3	Hardened Steel	75	260	0.93
PC	Polycarbonate	Polymaker	Artery Canulus, Blood Filter Housing, Various Apparti	Clear	4.6	Brass	80	270	0.82

### 3D printing parameters

The 3D printed devices were created using a fusion deposition modeling (FDM) additive manufacturing technique of polymer filaments. All objects were printed at a layer height of 0.2 mm to provide a balance between print time and vertical resolution with a 0.4 mm nozzle. For consistency, all objects were printed at a print speed of 50 mm/s for all features (infill, walls, and solid surfaces). An infill percentage of 35% with a rectilinear pattern was used, yielding solid mechanical strength while balancing printing time and material usage. Simplify3D v3.1.0 was used to generate toolpaths for all models.

Using recommendations provided by the material manufacturers and visual observations, the print quality for each material was optimized to determine ideal printing temperatures. Due to variances between batches, manufacturers, and die-swell for raw materials, the flow rate was optimized by printing a single walled object, measuring the actual wall thickness, and adjusting flow rate (extrusion multiplier) accordingly to match measured results with the predicted wall thickness. A summary of optimized printing temperatures, extrusion multipliers, and nozzle selection for each material are shown in Table 1. The hardened steel, stainless steel, and brass nozzles used for 3D printing were purchased from E3D (Chalgrove, Oxfordshire, UK) and were used only with the materials specified in Table 1 to avoid cross-contamination. In addition, before printing each test piece a minimum of 10 cm of material was extruded through the nozzle held at the temperature specified in Table 1 to purge the nozzle of any contaminants.

One set of the FDA-approved PLA (FDA PLA) devices was subjected to annealing by placing the printed materials on clean aluminium foil in a temperature controlled oven at 100°C for 18 minutes. The annealing procedure was conducted to allow for more complete crystallization of the polymer material. Polymer annealing can lead to higher values of tensional and flexural moduli of elasticity, Izod impact strength, and heat resistance of the polymer, which may be useful for certain medical device applications, such as orthopedic implants [27–29].

### Extraction procedure

Devices were extracted in three different solvents of varying polarity: water, isopropyl alcohol (IPA), and hexane. Each device was extracted using 50 mL of the pure solvent. Extraction was accomplished by adding a single device to an appropriate extraction vessel with the corresponding solvent and placed in an incubator shaker for >72 hours at 50°C, as recommended by ISO 10993–12 [30]. The incubator shaker was set to 100 rpm to ensure that the devices were completed wetted for the duration of the extraction. While there is currently no universal standard for medical device extraction procedures, several industry guidelines recommend using aggressive extraction conditions and a range of solvents that do not decompose the product [23, 31], which was taken into consideration for this study. The solvent volumes used were dictated by the minimum amount necessary for chemical analyses.

To provide a suitable comparison between printed and unprinted materials, the corresponding polymer filaments were extracted using the same procedures as the printed devices, except only 35 mL of solvent was used in the extractions.

The recovered extracts were tested using a suite of analytical techniques targeting volatile, semi-volatile, and low-volatility organics, in addition to elemental and particulate matter. All reagents were analytical grade purchased from Fisher Scientific, unless otherwise noted.

Extractions designed for elemental composition testing were conducted in polypropylene DigiTUBES (SCP Science) that were previously tripled rinsed with 1% nitric acid (HNO_3_, TraceMetal Grade, Fisher Scientific), while extractions designed for all other analytical testing were conducted in glass wide-mouth jars equipped with PTFE caps (FisherBrand, Fisher Scientific) that were triple rinsed with the solvent of interest prior to use. Elemental analysis extraction was only completed using water.

After extraction, the resulting samples were allowed to cool to room temperature before their contents were divided for analysis. For particulate analysis, 20 mL of the extract was used. For ICP analysis, 9.85 mL of the extract was transferred to a plastic recovery vessel and combined with 0.10 mL of HNO_3_ and 0.05 mL of HCl prior to analysis. For headspace GC/MS, 3.0 mL was directly transferred to glass headspace vials. For LC/MS and GC/MS analyses, the extracts were directly added to autosampler vials, with the exception of water analysis using GC/MS. For this analysis, 5 mL of the water sample was extracted in duplicate via liquid-liquid extraction in a separatory funnel with 5 mL dichloromethane (DCM). The resulting DCM extracts were combined and analyzed using GC/MS.

### Instrumental analysis

Static headspace (Perkin Elmer HS40XL) gas chromatography-mass spectrometry (GC/MS; Agilent 6890 and 5973, respectively) was used for volatile organic compound analysis of water extracts. A DB-624 capillary column was employed (30 m x 0.25 mm x 1.4 μm, Agilent) with an oven temperature program starting at 40°C for 2 minutes before a 15°C/minute ramp to 255°C. Semi-quantitation was accomplished by comparison to a decane (d_22_) external standard. All compound identifications in GC-MS analyses were made by comparison to the NIST library.

Direct inject GC/MS was used for semi-volatile organic compound analysis of all extracts. For the water extracts, a solvent-solvent extraction using dichloromethane was utilized prior to GC/MS analysis (see Extraction procedure). An XTI-5 capillary column was employed (30 m x 0.25 mm x 0.25 μm, Restek) with an oven temperature program starting at 40°C for 2 minutes before a 10°C/minute ramp to 310 °C. Semi-quantitation was accomplished by comparison to a hexadecane (d_34_) external standard.

Ultra-high performance liquid chromatography time-of-flight mass spectrometry (UPLC/TOF-MS; Waters H-Class UPLC and Xevo G2-XS QTOF, respectively) was used for low-volatility organic compound analysis of all extracts. A CORTECS C18 column was employed (100 x 2.1 mm, 1.6 μm, Waters, Corp.) with a flow rate of 0.4 mL/minute, and a 48 minute mobile phase gradient consisting of 2mM ammonium acetate in water and 2mM ammonium acetate in acetonitrile (ACN). The gradient was as follows: 10% ACN for 1 minute followed by an increase to 97% ACN in 17 minutes and a 25 minute hold before the gradient was decrease to 10% ACN and held for 5 minutes prior to the next injection. All solvent extracts were analyzed using the LC/MS technique. Semi-quantitation in both positive and negative mode was accomplished by comparison to a 2-mercaptobenzothiazole (98%, Sigma Aldrich) external standard. This standard was chosen because it had the lowest instrument response of all potential extractable compound classes tested, indicating that its use would produce an upper limit for extractable concentrations, in alignment with a worst-case-scenario approach. As this technique provided similar results across both GC-MS and LC-MS analyses for the extractable DEHP (see below), it was deemed suitable for this method. High resolution mass spectrometry data, along with the use of authentic standards, assisted in compound identification. Only compounds with concentrations above 5 μg/device were reported.

Inductively-coupled plasma mass spectrometry (ICP/MS; Thermo-Fisher XSeries2) was used for elemental matter analysis. Samples were acidified using HNO_3_/HCl prior to the analysis. Quantitative data was produced by comparison to an external standard for each element of interest (Cd, As, Hg, Pb, Ir, Os, Pd, Pt, Rh, Ru, Cr, Mo, Ni, V, and Cu). Elements were selected for analysis based on the 2015 revision of USP <232> [32].

Particulate matter analysis was accomplished using the light obscuration particle count test with a liquid particle counter (HIAC ROYCO, Pacific Sci.), following USP <788> Method 1 guidelines [33]. Only water and IPA extracts were analyzed for particulate matter, due to solvent compatibility concerns of the hexane extracts with the detector. Particle sizes distributions measured were ≥10 μm and ≥25 μm diameters. Chemical characterization of individual particles was investigated using a scanning electron microscope (SEM; FEI Quanta 200) equipped with an energy-dispersive X-ray detector (EDS; EDAX Pegasus EDS detector). IPA extracts were deposited onto a glass film connected to an aluminum sample holder via conductive carbon tape, and the solvent was allowed to dry before the samples were sputter coated with Pt to ~20 nm thickness (Quorum Q150RS). Printed device extracts from FDA PLA, FDA PLA annealed, PC, and PETG were analyzed, along with the extract from the control sample. EDS analysis was performed at 0.08 torr with a high voltage of 20 kV, a working distance of 11 mm, and a spot size of 4.

## Results

Following extraction, all device coupons (referred to as “devices” in this paper) were visually similar to pre-extraction observations. The devices showed no signs of swelling, regardless of the solvent used, and their morphology remained unchanged after extraction. The 3D printing process did have a notable effect on the chemical profiles and amount of particulate matter extracted from the devices, described in more detail below. Both the largest amount and number of organic compounds were observed in the IPA extracts, thus, only these results are displayed, unless where noted. The water extracts contained the smallest amount of observed components while the hexane extracts did not contain unique information compared to data from the IPA extracts. Since the devices studied were not created for a clinical use, the data generated herein provides an assessment of extractable components under controlled laboratory conditions rather than an evaluation of the specific impacts on patient safety.

### Volatile organics (headspace GC/MS)

Volatile organic compounds were not observed above the method detection limit (10 μg/device) in the static headspace GC/MS analysis of the water extracts from either the final devices or filaments. These results suggest that the printing process does not degrade polymers to the extent that volatile products are formed.

### Elemental analysis (ICP/MS)

Of the 15 elements targeted by ICP/MS analysis (see Experimental section), only lead (Pb) was observed in the printed device extracts. Pb was not observed above the method detection limit (0.35 ng/device) in the filament extracts. Results for elemental Pb in the final device extracts are shown in Table 2.

**Table 2:** The Pb concentrations observed in extracts of the original filaments and 3D printed devices, based on printing nozzle type used.

Material	Nozzle	Pb (ng/g)	Pb (ng/device)
PLA	Steel	0.13	0.69
FDA PLA	Brass	1.46	7.37
FDA PLA annealed	Brass	0.98	4.95
PETG	Steel	0.11	0.60
PC	Brass	1.08	4.95

The devices printed using the brass nozzle had 7–12 times greater concentrations of Pb compared to those printed using a steel nozzle. This correlation, along with the absence of Pb observed in filament extracts, provides evidence that Pb from a printing nozzle can contribute to the extractable profile of 3D printed materials.

### Particulate matter

**Fig 2:**
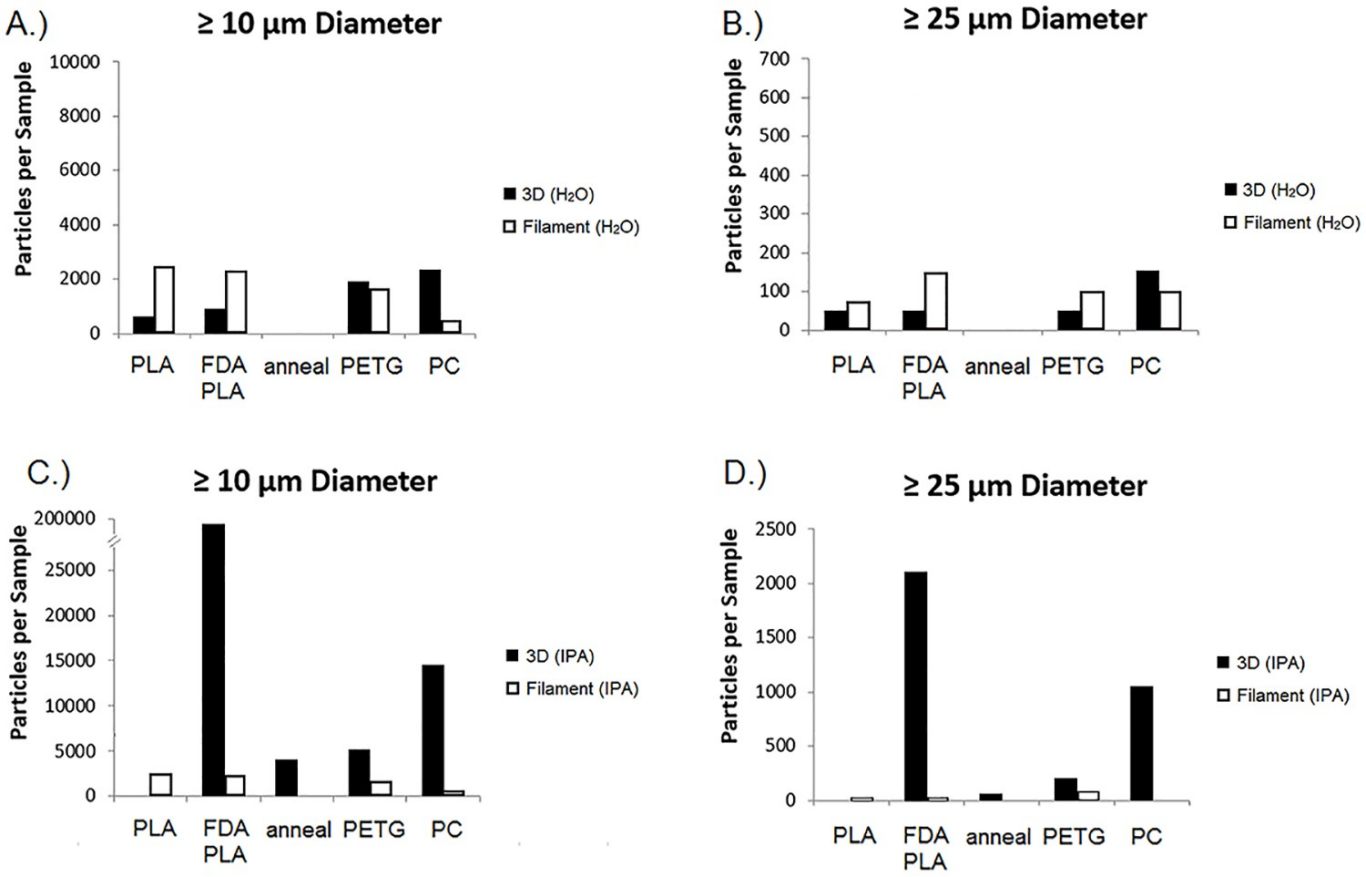
Particulate matter in extracts. The number concentrations of particles observed in each material extract for each given size distribution (≥ 10 μm and ≥ 25 μm). 3D printed device extracts are represented in black while the filament extracts are represented in white. The water extracts for each size distribution are displayed in A.) and B.) while the IPA extracts are displayed in C.) and D.). Note that the annealed extract was only available for the finished printed device testing of the FDA PLA polymer.

Both ≥10 μm and ≥25 μm sized particulate matter were observed in the filament and device extracts, as shown in Fig 2, which displays the particle number concentrations observed in each material extract. Fig 1A and 1B correspond to the water extracts for the ≥10 μm and ≥25 μm sizes, respectively, while Fig 1C and 1D correspond the IPA extracts for the ≥10 μm and ≥25 μm sizes, respectively.

The PC and FDA PLA extracts had the largest number concentrations of particulate matter, shown in Fig 2, with results from each respective IPA extract above the USP <788> Method 1 test compliance limit (>6000 counts per device and >600 counts per device for >10 μm and >25 μm testing, respectively) [33]. Overall, the total particle counts in the IPA extracts of the printed devices were 10 times greater than the particle counts in the corresponding filament extracts. In all cases, extraction with water did not lead to significant particle number counts. It is important to note that, since the extraction was not performed on medical devices designed for a specified treatment, the USP <788> testing limits do not correspond to safety thresholds for patient exposure and are provided only as a general reference.

Interestingly, the annealing process had a significant impact on the number of particles observed in the device extracts. Particulate matter in the extracts from the devices that underwent the post-printing annealing process had approximately 5000% less particles by number than those that were not annealed (see Fig 2). The annealing process was only applied to the FDA-approved PLA polymer devices. These results indicate that the crystallization of 3D printed materials is of great importance in relation to reducing particulate matter concentrations.

**Fig 3:**
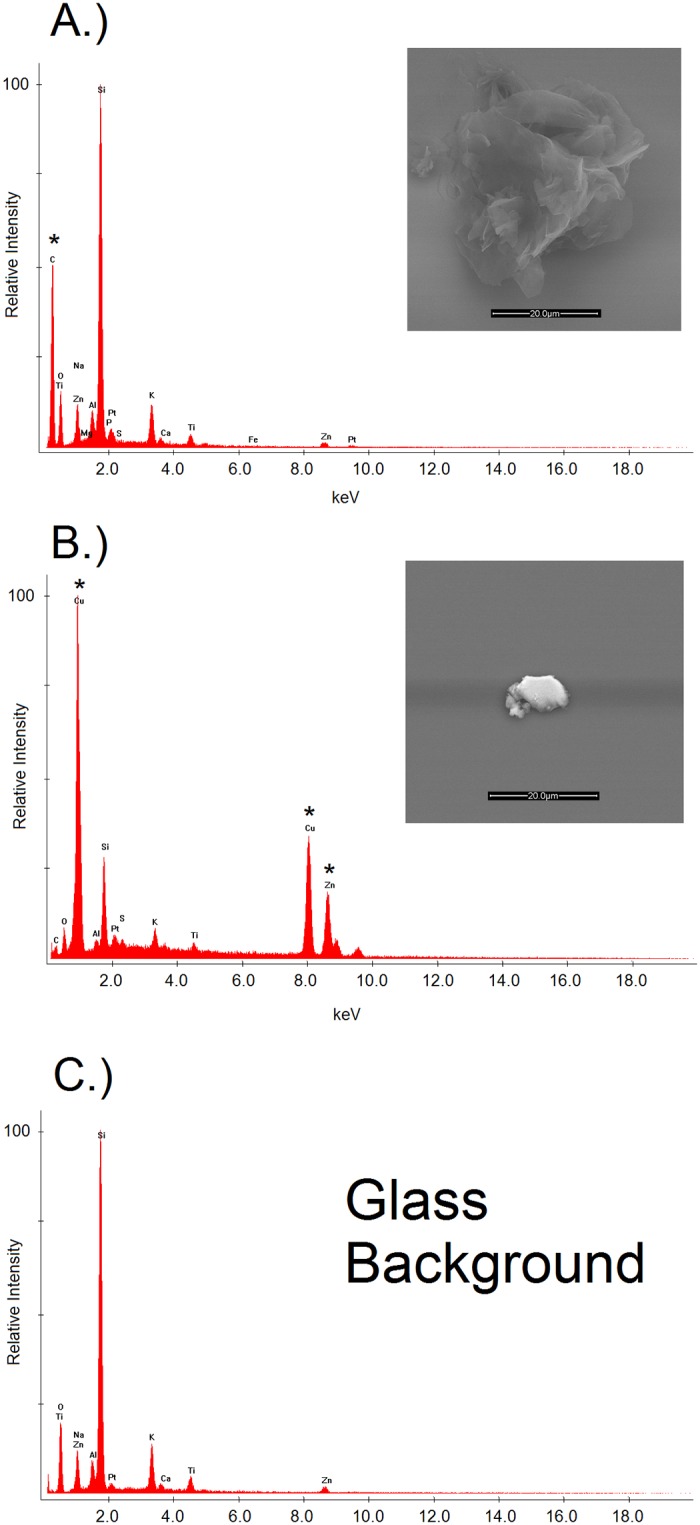
SEM-EDS analysis of particles. The SEM images and EDS elemental data for particles observed in the printed FDA PLA extracts. A polymer-derived particle is shown in (A.), as indicated by the C signal in the EDS spectrum. A metal particle originating from the brass printing nozzle is shown in (B.), as indicated by the Cu/Zn signals in the EDS spectrum. In the respective EDS spectra, an asterisk (*) denotes new peaks observed compared to the corresponding blank (C.).

Characterization using SEM-EDS revealed the amorphous physical structures and elemental compositions of individual particles. A representative example of the types of particles observed is shown in Fig 3. Additional figures displaying the SEM-EDS images and data for each extract tested are provided in the Supporting Information (S1–S3 Figs). While some particles contained Fe or Cu/Zn, corresponding to the steel and brass nozzles used for printing, respectively, a large majority of the extracted particles were derived from carbon, indicating that they were native to the polymer. Using the FDA PLA extract as a representative sample, only 2% of the observed particles contained metal related to the print nozzle.

### Semi-volatile organics (GC/MS)

Fifteen separate compounds were detected using the GC/MS technique. The polylactic acid precursor, L-lactide, was observed in all PLA and FDA PLA extracts, and had the largest measured concentrations of any analyte detected across all materials. The FDA-approved PLA devices had the largest amount of extractables organics, accounting for 84% of the semi-volatile organics observed by number in this study. Total analyte mass concentrations extracted from the printed and filament polymers were relatively similar, within 15% for each material. Complete tabulated results from the GC/MS analysis are shown in Table 3. Only compound identifications that had an MS match score against the NIST library greater than 70 were reported.

**Table 3:** The GC/MS results from each material extract in IPA.

Compound	Retention Time (min)	Device Amount (μg/g)					Filament Amount (μg/g)			
		PLA	FDA PLA	FDA PLA Annealed	PETG	PC	PLA	FDA PLA	PETG	PC
Isoprpyl lactate	4.8	--	--	--	--	--	--	11	--	--
DL lactide	9.4	7	12	5	--	--	5	8	--	--
L-Lactide	10.0	217	450	270	--	--	167	399	--	--
m/z 59, 117, 131, 173	20.0	--	26	16	--	--	--	6	--	--
m/z 56, 116, 170, 184	23.0	--	23	10	--	--	--	26	--	--
m/z 58, 117, 175	23.3	--	27	17	--	--	--	60	--	--
m/z 32, 128, 145, 200, 272	23.4	--	10	--	--	--	--	13	--	--
Triphenyl phosphate	23.4	--	--	--	--	--	--	--	--	3
DEHP	24.5	--	6	--	--	--	--	4	--	--
m/z 56, 128, 200, 272,344	25.8	--	8	--	--	--	--	8	--	--
Unknown siloxane	26.2	--	30	17	--	--	--	8	--	--
Unknown siloxane	27.8	--	--	--	--	--	--	--	22	--
Unknown siloxane	28.6	--	31	25	--	--	--	43	--	--
Unknown siloxane	29.7	--	--	--	--	--	--	--	6	--
Unknown siloxane	31.2	--	27	9	--	--	--	8	--	--

Data from the 3D printed device extracts and filaments are given in μg/g. Siloxanes were determined based on their distinct fragmentation patterns.

Similar to the particulate matter analysis, a decrease in concentration was observed in the annealed FDA PLA device extracts compared to those from the untreated FDA PLA device. The total mass concentration of analytes detected in the annealed devices was 43% less than the concentrations observed in the untreated devices. Additionally, while DEHP was observed at similar concentrations in both the FDA PLA printed device and filament extracts, it was not observed above the method’s detection limit (5 μg/device) in the annealed device extracts, indicating that–in addition to physical characteristics–the annealing process also had an impact on the chemical signature of the material. One possible explanation for this observation is that the printing procedure induces a thin film of DEHP on the surface of the device, which could lead to evaporation at temperatures as low as 100°C [34].

### Low-volatility organics (LC/MS)

**Fig 4:**
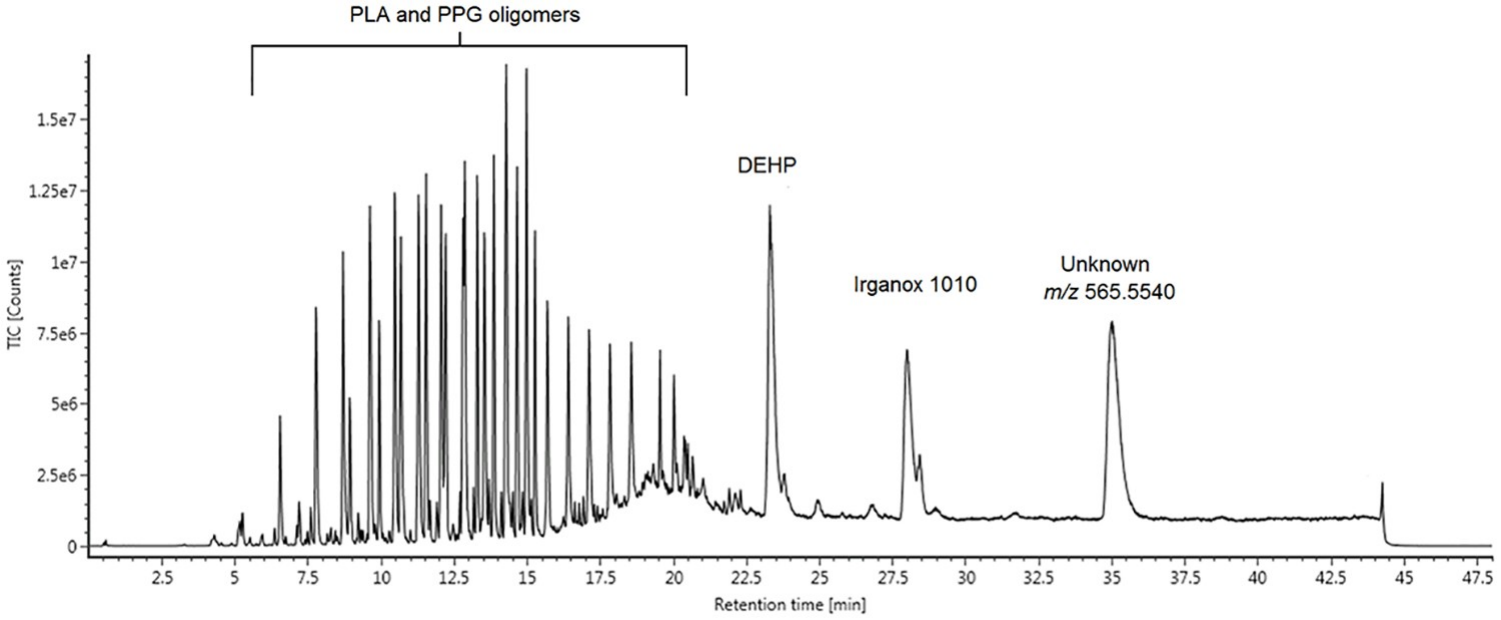
LC/MS chromatogram. The LC/MS total ion current chromatogram (TIC) of an FDA-approved PLA 3D printed device extracted in IPA.

A representative total ion current chromatogram (TIC) from the LC/MS analysis of a device extract is shown in Fig 4. The main class of organic compounds detected was the oligomers derived from the PLA polymers. PLA is a biocompatible polymer that hydrolyzes easily [35], thus, it was not a surprise to see a large number of PLA oligomers in the extracts. More surprising was the presence of polypropylene glycol (PPG), a co-polymer of PLA, in the FDA-approved PLA material extracts, as this was not listed as a material component from the manufacturer. The PPG-derived oligomers accounted for 72% of the total oligomers detected from the FDA-approved PLA material. No oligomeric species were detected in either the PETG or PC extracts.

**Fig 5:**
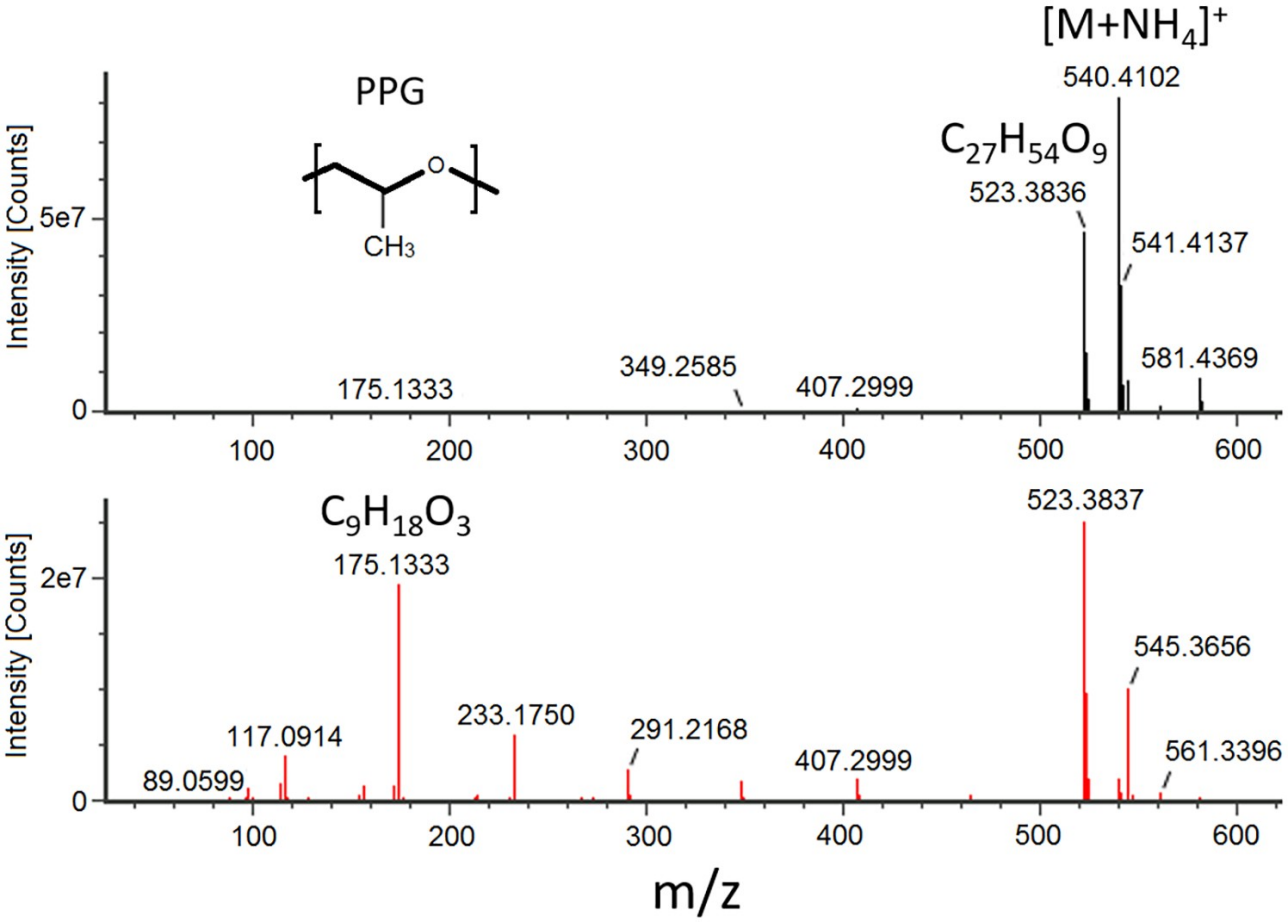
Oligomer mass spectra. The low energy (top) and high energy (bottom) mass spectra of a C_27_H_54_O_9_ PPG oligomer extracted from an FDA-approved PLA device in IPA. Fragments in the high energy spectra show separation by *m/z* 58.042, the mass of a C_3_H_6_O PPG monomer.

**Fig 6:**
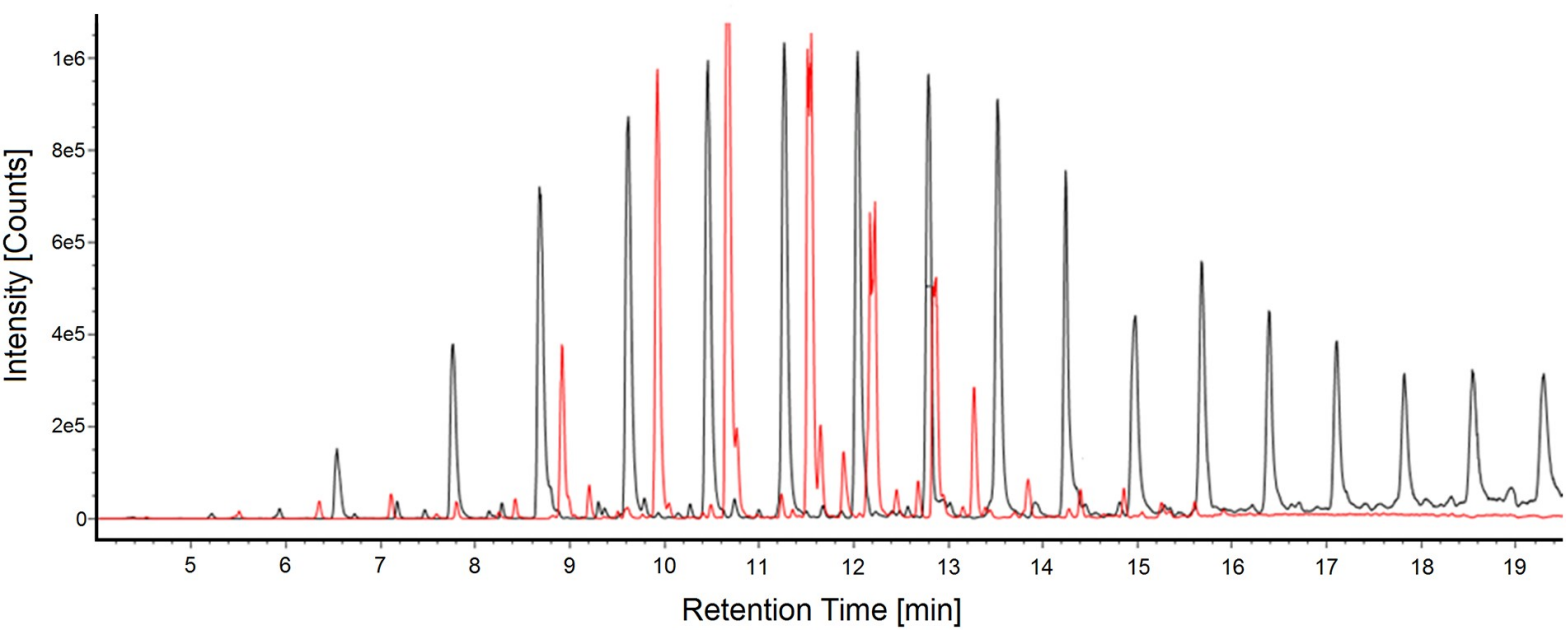
Oligomer EICs. The extracted ion current chromatograms (EICs) of the PPG trimer (*m/z* = 175.1330; black trace) and the PLA trimer (*m/z* = 217.0710; red trace) from an FDA PLA device extract in IPA.

Representative mass spectra for a PPG oligomer are shown in Fig 5. Along with fragmentation displaying a distinct loss of monomer units, a trimer fragment in the high energy mass spectra for the PPG co-polymer (*m/z* 175.1330) was consistently observed and, thus, could be used as a tracer for the oligomer. Similar mass spectra patterns were observed for the PLA oligomers, with the PLA trimer (*m/z* 217.1010) present in all high energy mass spectra. An extracted ion current chromatogram (EIC) for each respective trimer fragment ion in the high energy spectra is shown in Fig 6, displaying a near Gaussian-like distribution of the PLA and PPG oligomer intensities with even spacing between observed peaks. This distinct chromatographic pattern further supports the presence of an oligomeric species. Calculated concentrations for each oligomeric species observed are given in Tables 4 and 5.

**Table 4:** The PLA oligomers extracted from each listed material using IPA.

Retention Time (min)	PLA Oligomer	Observed mass	Mass error (ppm)	ESI(+) Adducts	Amount (mg/sample)				
					PLA Filament	PLA Device	FDA PLA Filament	FDA PLA Device	FDA PLA Annealed
9.33	C15H20O10	360.1044	-1.8	+NH4, +K, +Na	5	7	22	16	12
10.34	C18H24O12	432.1256	-1.4	+NH4, +H, +K, +Na	8	11	22	17	13
11.09	C21H28O14	504.1473	-0.1	+NH4, +H, +K, +Na	12	15	28	12	16
11.95	C24H32O16	576.1688	0.6	+NH4, +K, +Na	17	19	32	13	18
12.61	C27H36O18	648.1892	-0.6	+NH4, +K, +Na	21	20	39	15	22
13.27	C30H40O20	720.2100	-0.9	+NH4, +K, +Na	17	19	35	17	20
13.69	C33H44O22	792.2318	-0.1	+NH4, +K, +Na	14	17	34	15	19

PLA oligomers detected using an LC/QTOF-MS technique.

**Table 5:** The PPG oligomers extracted from each FDA PLA material using IPA.

Retention Time (min)	PPG Oligomer	Observed mass	Mass error (ppm)	ESI(+) Adducts	Amount (mg/sample)		
					Filament	Device	Annealed
6.6	C15H30O5	290.2077	-3.8	+H, +K, +Na, +NH4	17	12	11
7.8	C18H36O6	348.2496	-3	+H, +K, +Na, +NH4	26	17	16
8.7	C21H42O7	406.2915	-2.4	+NH4, +H, +K, +Na	31	23	21
9.6	C24H48O8	464.3340	-0.7	+NH4, +H, +K, +Na	38	26	24
10.5	C27H54O9	522.3761	-0.1	+NH4, +H, +K, +Na	40	27	11
11.3	C30H60O10	580.4179	-0.3	+NH4, +H, +K, +Na	41	28	26
12.0	C33H66O11	638.4595	-0.7	+NH4, +H, +K, +Na	39	28	25
12.8	C36H72O12	696.5020	0.3	+NH4, +H, +K, +Na	35	26	23
13.5	C39H78O13	754.5432	-0.6	+NH4, +H, +K, +Na	37	27	23
14.2	C42H84O14	812.5852	-0.4	+NH4, +H, +K, +Na	31	21	19
15.0	C45H90O15	870.6273	-0.1	+NH4, +H, +K, +Na	28	19	17
15.7	C48H96O16	928.6691	-0.1	+NH4, +H, +K, +Na	27	19	15
16.4	C51H102O17	986.7111	0	+NH4, +H, +K, +Na	22	16	12
17.1	C54H108O18	1044.7538	0.8	+NH4, +H, +K, +Na	19	13	10
17.8	C57H114O19	1102.7959	0.9	+NH4, +H, +K, +Na	15	10	7
18.6	C60H120O20	1160.8368	0.1	+NH4, +H, +K, +Na	13	16	11

PPG oligomers detected using an LC/QTOF-MS technique.

While the concentration of oligomeric species observed in the annealed FDA PLA device extracts did not differ significantly from the concentrations observed in the untreated FDA PLA extracts, the oligomer concentrations in the filament extracts of both the PLA and FDA PLA materials were greater than the respective printed device extracts. The filament extracts had a 25% greater oligomer concentration in the PLA material and a 171% greater oligomer concentration in the FDA PLA material compared to the device extracts. The larger concentration of hydrolyzable oligomers observed in the filament extracts may be related to the greater surface area to volume ratio of the filaments (32.2 cm^2^/cm^3^) compared to the devices (5.2 cm^2^/cm^3^).

Several other compounds were also detected in the LC/MS analysis, as shown in Table 4. Most notably, the antioxidant Irganox 1010 and the plasticizer diethylhexyl phthalate (DEHP) were observed in the FDA-approved PLA extracts. While DEHP is a common plasticizer often found in laboratory settings, it was observed at a concentration more than three times that of the background in all reported instances. In contrast to the FDA-approved material, no polymer additives were observed in any PLA extract. The main extractable observed from the PETG was the colorant, Disperse Yellow 3G, while two phenyl phosphates were observed in the PC material extracts. The semi-quantitative calculated concentrations of each detected extractable are given in Table 6.

**Table 6:** Non-oligomeric compounds detected in LC/QTOF-MS analysis of the IPA extracts.

Retention Time (min)	Compound	Observed Mass	Adduct	Mass error (ppm)	Device Amount (μg/g)					Filament Amount (μg/g)			
					PLA	FDA PLA	Anneal	PETG	PC	PLA	FDA PLA	PETG	PC
12.0	Triphenyl phosphate	327.0789	+H	-1.0	--	--	--	--	3	--	--	--	9
12.3	Latyl Yellow 3G	290.0804	+H	1.8	--	--	--	137	--	--	--	140	--
16.6	Bisphenol-A bis(diphenyl phosphate)	693.1842	+H	-5.0	--	--	--	--	83	--	--	--	179
19.0	DEHP	391.2848	+H	-0.3	--	5	4	--	--	--	4	--	--
23.3	Irganox 1010	1194.8251	+NH_4_	-0.6	--	1232	306	--	--	--	1415	--	--
35.0	C_36_H_72_N_2_O_2_	565.5540	+H	1.1	--	1268	586	--	--	--	--	--	--

In comparison to the filament extracts, most organic compounds were found to have similar concentrations in the corresponding device extract, with two notable exceptions. The first being an unknown compound with the assigned chemical formula C_36_H_72_N_2_O_2_ (*m/z* 565.5640; see Table 6) that was observed in both the PLA and FDA PLA device extracts. Since the unknown was only observed in the final device extracts, the compound is believed to be an artifact of the printing process. This unknown did not fragment up to a collision energy of 45 eV, thus, structural information about the compound could not be ascertained. However, the late elution of the unknown compound in the LC gradient indicates that it is relatively hydrophobic, meaning it could contain alkane functionality. The printing process also had an impact on the amount of phenyl phosphates observed in the PC device extracts. A 55% smaller concentration of phenyl phosphates was observed to migrate from the printed devices compared to the filaments. This is likely related to the evaporation of the additives during the printing process, which has been observed for triphenyl phosphate at temperatures above 200 °C [36].

## Discussion

This study provides chemical information on the extractables that can arise from 3D printed products. Of the extractables, the compound with the most notable public health risk observed, DEHP [37], migrated from the FDA-approved PLA device at 6 μg/g, which would correspond to a total of 30 μg for a 5 g device (see Table 3). Under a worst-case-scenario assumption where all of the DEHP is released in a single day, a 4 kg neonate–the highest risk patient population–would theoretically be exposed to a maximum of 7.5 μg DEHP/kg/d. For comparison, the lowest tolerable daily intake published by the US FDA is 40 μg DEHP/kg/d [38]. Although, since this value is based on lifelong continuous oral exposure, it is important to note that the comparison presented is for reference only and does not represent an assessment of patient risk.

One potential concern was the unknown compound observed in both the PLA and FDA-approved PLA extracts originating from the 3D printing process. With a measured concentration of extractable mass at ~6 mg per device (assuming a 5 g device), the unknown compound is at a level well above the threshold of toxicological concern for genotoxic impurities outlined by the FDA (1.5 μg/day) under worst-case-scenario conditions [39]. Thus, depending on the medical device application and material, the FDM printing process may produce products of higher risk than previously assumed.

Along with potential chemical concerns, the large number of particulates observed in the FDA-approved polylactic acid (FDA PLA) and polycarbonate (PC) device extracts are also of interest. While the particulate matter concentrations in water extracts for each material were relatively small, the mid-polar solvent (IPA) extracts of the FDA PLA and PC devices both had number concentrations more than 3 times those from any other material. In the case of the extracted FDA-approved PLA device, the ≥10 μm number concentration (208,000 mL^-1^) was more than 14 times greater than the next largest observed number concentration (14,400 mL^-1^ from PC), as displayed in Fig 2. The observed particle number concentrations of the FDA PLA device extracts decreased significantly when the devices were subjected to an annealing process, dropping to 4,050 mL^-1^. Printing via the FDM process led to 10-fold greater particle concentrations in device extracts compared to the extracts of the original filaments. While metallic particles were observed that originated from the printing nozzles, the vast majority of the particles characterized were derived from the polymer itself. Future work should investigate the effects of post-processing and sterilization procedures on the extractable profiles of final marketed devices.

In addition to the largest concentration of particles observed, the extracts from the FDA-approved PLA printed devices also had the largest concentration of total extractable chemicals. Specifically, in contrast to the PLA polymer, extracts of the FDA PLA material contained a plasticizer (DEHP), an antioxidant (Irganox 1010), and a co-polymer (PPG). Thus, use of the FDA-approved PLA should be associated with the most caution out of all the materials studied when considering extractables in a 3D printed medical device design.

## Conclusions

Based on the results of this study, the application of an annealing procedure to 3D printed medical devices can be used to greatly reduce the complexity of its extraction profile. An annealing procedure applied to final devices in this study led to a decrease in extractable semi-volatile organic compound concentrations by 43%, which included a phthalate plasticizer (DEHP), and particulate matter concentrations by a factor of 50. In addition, the large amount of an unknown compound compared to other extracted compounds from the PLA and FDA PLA manufactured devices indicates that the FDM printing process may alter the chemical profiles of polymeric material. The FDM printing process also led to 10 times greater particulate matter concentrations in the IPA extracts of final printed devices compared to the original filaments. Of the materials tested, the FDA-approved polylactic acid polymer extracts had by far the largest concentrations of chemical constituents and particulate matter observed, meaning it should be used with the most caution of all the materials studied. In addition to high levels of particulate matter originating from the FDA PLA and polycarbonate 3D printed devices, the results also indicated that the use of brass printing nozzles and material colorants can lead to artifacts during the device manufacturing processes.

## Supporting information

S1 Fig Polyethylene terephthalate-glycol SEM-EDS. SEM images and EDS elemental data for particles observed in the IPA extract of printed polyethylene terephthalate-glycol devices. The image in (A.) corresponds to a particle originating from the stainless steel printing nozzle, as indicated by the large Fe peak in the EDS spectrum. The image in (B.) corresponds to a particle derived from the polymer, as indicated by the C peak in the EDS spectrum.
(TIFF)

S2 Fig FDA PLA annealed SEM-EDS. An SEM image and EDS elemental data for a C-containing particle observed in the IPA extract of printed and annealed FDA-approved polylactic acid.
(TIFF)

S3 Fig Polycarbonate SEM-EDS. An SEM image and EDS elemental data for a C-containing particle observed in the IPA extract of printed PC.
(TIFF)
