# Supplementary figures and images for: Identifying extractable profiles from 3D printed medical devices
Source: PLoS One. 2019 May 22;14(5):e0217137. doi: 10.1371/journal.pone.0217137 (PMC6530847; doi:10.1371/journal.pone.0217137)

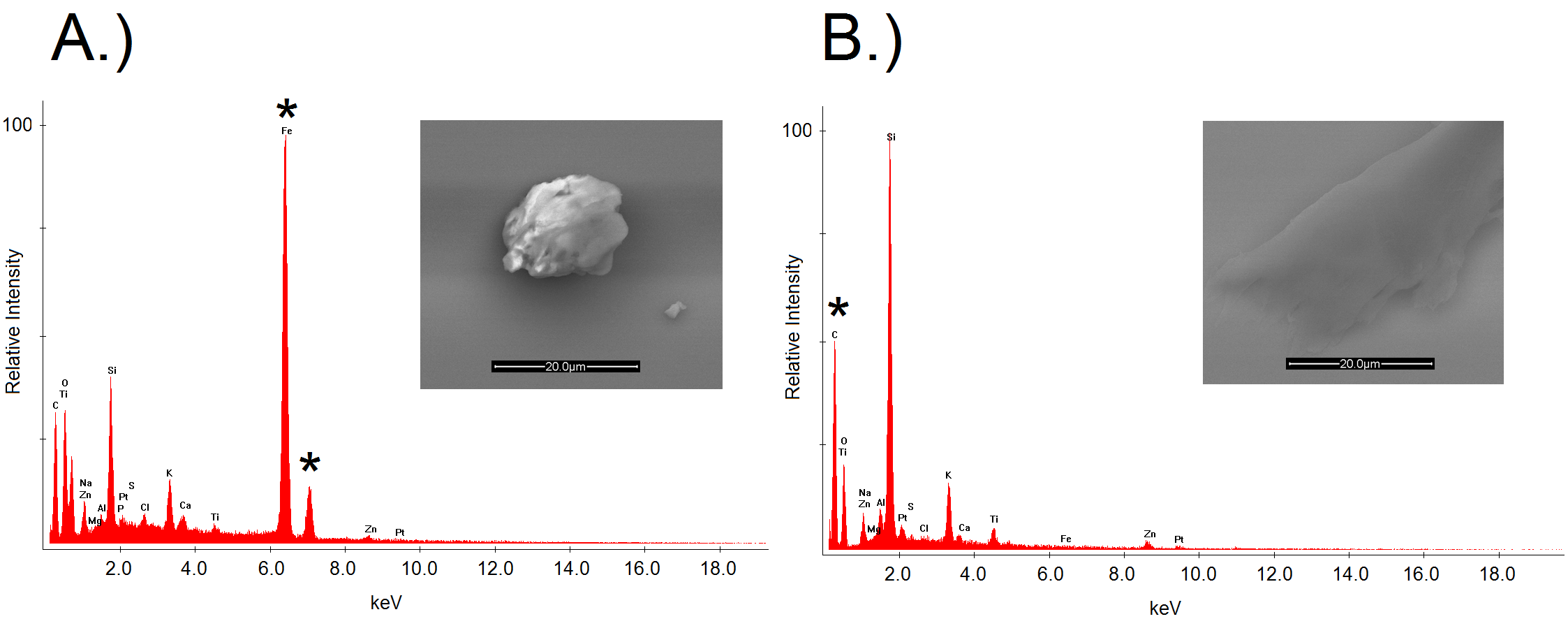

Supplement: S1 Fig — SEM images and EDS elemental data for particles observed in the IPA extract of printed polyethylene terephthalate-glycol devices. The image in (A.) corresponds to a particle originating from the stainless steel printing nozzle, as indicated by the large Fe peak in the EDS spectrum. The image in (B.) corresponds to a particle derived from the polymer, as indicated by the C peak in the EDS spectrum. (TIFF) [file pone.0217137.s001.tiff]

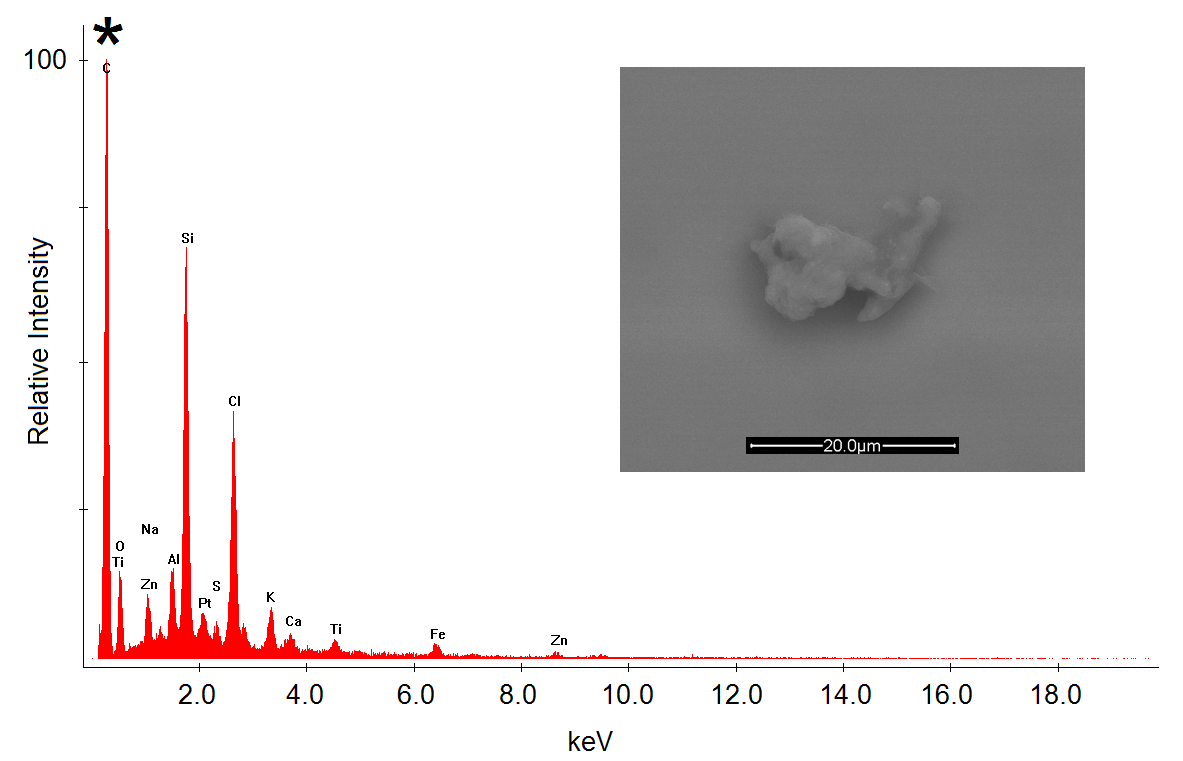

Supplement: S2 Fig — An SEM image and EDS elemental data for a C-containing particle observed in the IPA extract of printed and annealed FDA-approved polylactic acid. (TIFF) [file pone.0217137.s002.tiff]

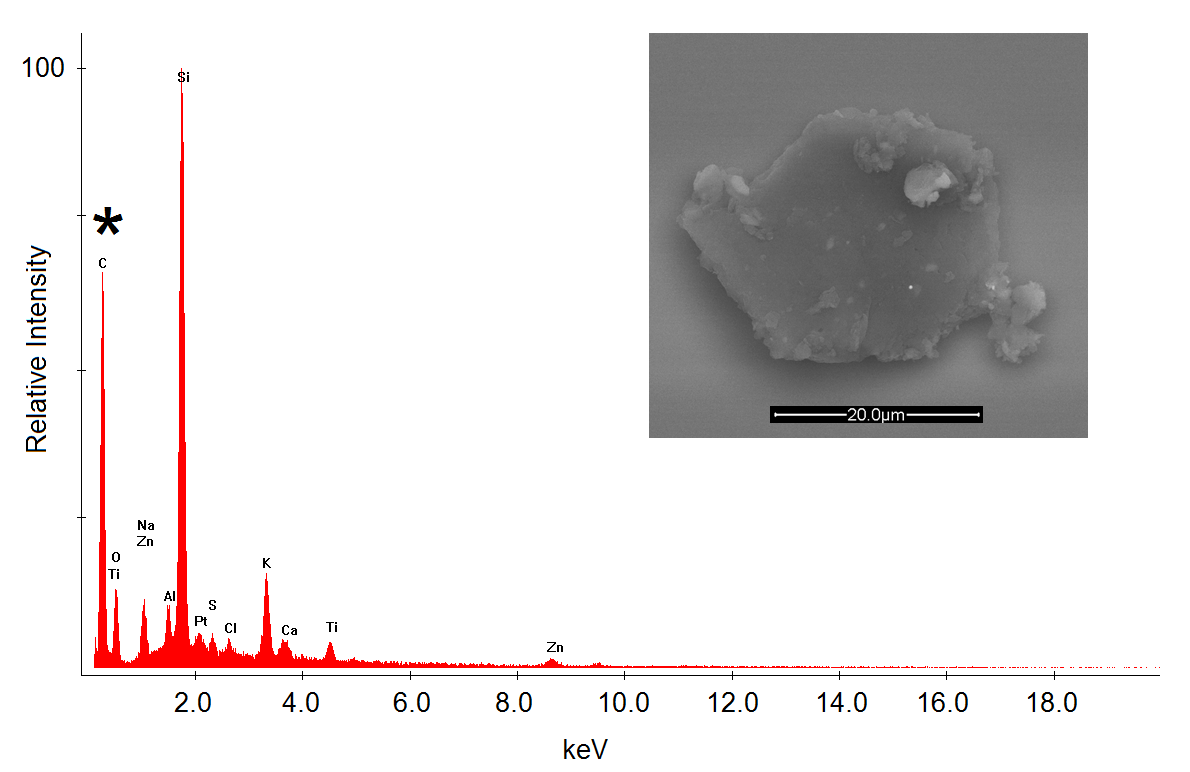

Supplement: S3 Fig — An SEM image and EDS elemental data for a C-containing particle observed in the IPA extract of printed PC. (TIFF) [file pone.0217137.s003.tiff]
